# The analytic hierarchy process method to design applicable decision making for the effective removal of 2-MIB and geosmin in water sources

**DOI:** 10.1007/s11356-024-31848-7

**Published:** 2024-01-17

**Authors:** Cihan Ozgur

**Affiliations:** Isparta University of Applied Sciences, Sutculer Prof. Dr. Hasan Gurbuz Vocational School, Isparta, Turkey

**Keywords:** 2-MIB, AHP, Drinking water, Geosmin, Treatment

## Abstract

Numerous utilities encounter issues with taste and odor that alter the public’s impression of the safety of drinking water. The creation of certain components in water naturally due to global climate change is another source of taste and odor components, in addition to industrial emissions. Geosmin and 2-methylisoborneol (2-MIB), both of which are generated by blue-green algae and actinomycetes, are two substances that contribute to the musty and earthy smells in drinking water sources. Unfortunately, current conventional treatment plants only partially remove 2-MIB and geosmin. Therefore, to protect the environment and public health, more up-to-date or optimized treatment methods should be applied to outdated treatment facilities. Best treatment practices, evaluation standards, and decision-making approaches, however, are still shrouded in mystery. The goal of this study was to identify the most effective treatment options for 2-MIB and geosmin. By using the analytical hierarchy process (AHP), a total of 22 assessment criteria were found and prioritized. A thorough literature search led to the identification of potential treatment options, and their effectiveness was evaluated. These options and priority rankings were decided upon using AHP in the decision-making process. Advanced oxidation techniques came out on top in the final priority ranking, followed by membrane filtering, adsorption, oxidation, hybrid processes, and traditional treatment methods. The applied analytical decision techniques may also be used to choose the optimal treatment options, even though the results are particular to 2-MIB and geosmin.

**Supplementary Information:**

The online version contains supplementary material available at 10.1007/s11356-024-31848-7.

## Introduction

Natural water resources produce taste and odor (T&O) as a result of hazardous cyanobacterial bursts caused by eutrophication and global warming (Manning and Nobles 2017). Many people wrongly think that smells are merely a question of taste, but smell is the primary indicator of a drinkable water’s safety (Wu et al. 2022). Cyanobacterial secondary metabolites, such as 2-methylisoborneol (1,2,7,7-tetramethyl-exo-bicyclo-[2,2,1]-heptan-2-ol, MIB) and geosmin (trans-1, 10-dimethyl- trans-9-decalol), have been identified as the primary musty/earthy odorants responsible for the widely observed T&O concerns in source water (Mustapha et al. 2021). These chemicals provide musty and earthy off smells and are semi-volatile in nature (Greenwald et al. 2015). Geosmin and 2-MIB have been found to have odor threshold concentrations that range from 4 to 10 and 9 to 42 ng/L, respectively (Soyluoglu et al. 2022). Globally, there is a growing need for T&O management in drinking water (Suruzzaman et al. 2022). Water consumers are now worried about the quantity of T&O compounds found in lakes and reservoirs (Bhateria and Jain 2016). Odor serves as an indicator for the presence of contamination; however, water without a discernible odor may not necessarily be safe for consumption. Moreover, certain pollutant odors can be detectable even in quite a small quantity (Sharma and Bhattacharya 2017). The traditional treatment methods used in water treatment facilities (such as coagulation, sedimentation, filtration, and disinfection) are particularly ineffective in removing 2-MIB and geosmin, which can cause drinking water to smell of dirt or mildew and fail to fulfill drinking water regulations (Srinivasan and Sorial 2011).

Advanced oxidation, chemical oxidation, biodegradation, and activated carbon adsorption are now effective methods for removing odors (Antonopoulou and Konstantinou 2017). However, excessively high odor concentrations significantly reduce the efficacy of activated carbon’s adsorption (Johnson et al. 2014). A lengthy treatment cycle is also required by biodegradation, and traditional oxidants like chlorine dioxide have poor removal efficiency (Liang et al. 2021). Advanced oxidation technology has attracted a lot of interest because of its advantages, which include powerful free radical oxidation potentials, low selectivity, rapid reaction rates, and simple conditions (Rekhate and Srivastava 2020). Through advanced oxidation, many organic pollutants that are difficult to break down can be successfully eliminated (Tufail et al. 2020).

Choosing the right treatment process is a crucial aspect when planning and implementing a water treatment plant (WTP). Multi-criteria decision-making (MCDM) techniques play a vital role in structuring the problem systematically. This characteristic empowers decision makers to thoroughly examine and tailor the problem according to their specific requirements (Isiklar and Buyukozkan 2006). The Analytical Hierarchy Process (AHP) proves valuable in managing multiple criteria and objectives during the decision-making process. Notably, the application of AHP allows the consideration of socio-cultural and environmental objectives, recognizing their equal importance alongside economic objectives in selecting the optimal water treatment alternative (Ellis and Tang 1991 and 1994). The AHP approach, as a systematic analysis technique for MCDM, facilitates a rigorous definition of priorities and preferences for decision makers. It aids in determining the weights of various factors (Saaty 1977, 1988; Cheng and Wang 2004; Bandyopadhyay and Chattopadhyay 2007). Conventional methods for process selection fall short in addressing the imprecise or vague nature of linguistic assessments.

Numerous studies in the literature have employed AHP methods to address various MCDM problems. Initially introduced by Saaty (1988), the AHP method has proven to be effective in solving MCDM problems and has found widespread application in successfully addressing practical challenges. The AHP approach, as a systematic analytical technique for MCDM, allows decision makers to establish clear priorities and preferences (Saaty 1977, 1990a, b; Guangming et al. 2007). AHP serves as a powerful and flexible MCDM tool, particularly useful for complex problems requiring consideration of both qualitative and quantitative aspects. By organizing critical aspects of a problem into a hierarchy resembling a family tree, AHP aids analysts in ranking decision alternatives to select the most optimal one when faced with multiple criteria (Taylor 2009). While traditional AHP relies on exact or crisp judgments, real-world decision problems often involve complexity and uncertainty, making decision-makers hesitant to provide precise judgments. Additionally, the subjective nature of individual judgments and varying interpretations of the same words poses challenges (Soner Kara and Onut 2010; Zhao et al. 2011). AHP’s applicability extends to environmental and social objectives, recognized as equally important as economic objectives in selecting the optimal water treatment alternative. Extensive literature addresses scenarios where comparison ratios involve imprecise judgments (Leung and Chao 2000). In real-world problems, some decision data can be precisely assessed, while others cannot. Human proficiency lies in qualitative forecasting rather than quantitative predictions (Kulak and Kahraman 2005). Some limitations identified by Srdjevic et al. (2012) regarding AHP are explained as follows. While the AHP methodology is widely recognized by the global scientific community as a resilient and versatile tool for multicriteria decision-making in addressing intricate decision problems, it does exhibit certain limitations. Notably, a significant drawback arises from the challenge of dealing with uncertainty, particularly prevalent in environmental issues. This uncertainty is introduced through the preference matrix, which incorporates pair-wise comparisons involving subjective and uncertain factors. Another constraint in the application of AHP pertains to real-world problems that defy hierarchical structuring, as they entail intricate interactions and dependencies between the highest and lowest elements. Tomar and Borad (2012) used the AHP method to determine the weights and main evaluations of the performance of seven performance parameters in their completed study. AHP connections are very useful and reliable. Evaluating the use of the AHP method, it was concluded that these weights could help in efficiency analysis by determining distributions at more careful intervals based on priority.

The goal of this study is to apply an analytical hierarchy process (AHP) to identify the best treatment options that may be employed to modernize the T&O removal of drinking water sources. These are the primary justifications for selecting AHP: (i) based on multiple techniques and viewpoints in choosing the best alternative, (ii) readily applicable and helpful, (iii) intelligible and interpretable in the findings produced, (iv) widely utilized, and (v) have not been used to discover the best treatment choices for the elimination of 2-MIB and geosmin. Numerous investigations on 2-MIB and geosmin’s capacity to be treated on a laboratory scale have been conducted by researchers. However, engineers, operators, and other decision-makers cannot assess treatment possibilities based only on the outcomes of laboratory-scale tests. The focus of this work is on 2-MIB and geosmin, two of the specified T&O compounds, both of which have distinct physical and chemical characteristics. The primary justifications for using AHP are as follows: (i) to find the best options based on many ways and viewpoints, (ii) simple to apply and helpful, (iii) the results obtained are intelligible and interpretable, (iv) widely distributed, and (v) many research on treatment did not utilize it to discover the best options for removing 2-MIB and geosmin.

The originality of this study is in choosing the optimal 2-MIB and geosmin removal treatment options by AHP. Regarding the techniques employed, applied methodology, assessments, and outcomes, this study can be helpful to other researchers, project engineers, plant operators, and other decision-makers. Furthermore, it can provide a roadmap for deciding on the optimal treatment solutions during the modernization process of traditional treatment facilities.

### AHP as a decision-making method

**Fig. 1:**
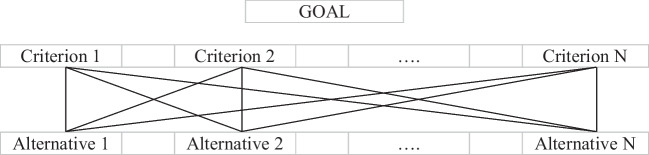
The main framework of the study

According to Thomas Saaty, who developed the AHP in the 1970s, the AHP for decision-making is based on a theory of relative measure that compares pairs used for standardized tables of absolute numbers, the components of which are then utilized as priorities (Saaty and Kulakowski 2016). Because it is a multicriteria method, it should represent the priority of the possibilities acquired under the various criteria (Saaty 2006). As a consequence, the AHP aids decision-making through pairwise comparisons of preset criteria evaluated by professionals (Saaty 2008). The important and critical criteria are chosen and arranged using a hierarchy structure that descends from a general goal through criteria, sub-criteria, and options in successive tiers (Saaty 1990a, b). The main framework of the study is shown in Fig. 1.

According to Saaty (2008), the decision-making process should be organized and separated into four steps: (1) identify the problem and the kind of data needed; (2) structure the decision hierarchy such that the choice goal comes first, then the objectives from a broad perspective, then the intermediate level (criteria on which the subsequent items depend), and lastly the lowest level (usually a group of possibilities); (3) compile a set of matrices for pairwise comparisons. The elements in the top level are individually used to compare the ones in the level directly below. After that, using the weights produced from the comparisons in step four, the priorities at the level below are prioritized. Each component is treated in this way. Then, to determine each element’s total global priority, sum the weight values for each level below. Continue adding and weighing possibilities until the final priorities of the options at the bottommost level are established. The realization and analysis of decisions are performed by the AHP using decision square matrices of *n* orders and the related eigenvectors. In Table 1, the AHP is illustrated using the largest calculated eigenvalue. Due to the possibility that it represents the level of consistency in judgment, this score is noteworthy. Matrix consistency only exists if > *n*. The consistency of the comparison’s matrix was assessed using a consistency index (CI), which was created. A random index (RI value) is influenced by the size of the parity comparison matrix. If the confidence interval is 0.1, the consistency of the comparison matrix will be acceptable; otherwise, the judgment may be changed (Saaty 1990a, b). The AHP has been applied across many fields and throughout the world.

**Table 1:**
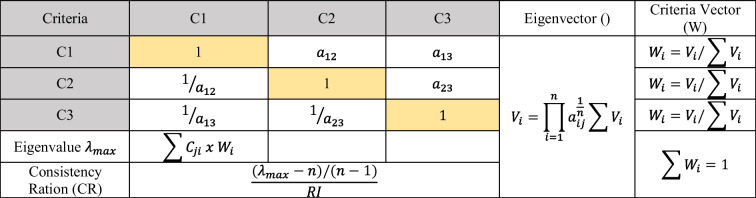
A pairwise comparison matrix

## Material and methods

There are typically three fundamental stages to this investigation. The assessment criteria for the preliminary phase were established based on the resources available and any specialized or expert knowledge. These evaluation criteria were chosen above others because they encompass a significant portion of the operational, technical, economical, and environmental factors taken into account when choosing a treatment procedure. Face-to-face interviews and a questionnaire were used to get the opinions of the experts. The assessment criteria were organized by AHP using expert comments as well. The second phase included a thorough review of the literature and the selection of several treatment methods for removing 2-MIB and geosmin from water sources. They were also weighted based on a number of assessment factors. AHP is a hierarchical, subjective multi-criteria decision-making (MCDM) process. The AHP methodology is composed on three core ideas. The framework of the model comes first, then a comparison of the alternatives and criteria, and ultimately, priority-setting. A pairwise comparison matrix, shown in Table 1, is used to evaluate a set of *n* criteria in accordance with their relative relevance weights. The format of the questionnaire list used is shown in Appendix 1.

Removal performance (C1), reliability and durability (C2), simplicity (C3), ease of planned maintenance (C4), ease of operation and routine maintenance (C5), ease of construction (C6), usage of various chemical (C7), major operational consumables (C8), proven/establishment technology (C9), generated wastes (C10), treatment/management requirements of wastes (C11), water efficiency (C12), security of supply (C13), asset life (C14), availability of technology (C15), pretreatment requirements (C16), by product/metabolite formation (C17), suitability of application (C18), additional treatments (C19), environmental impacts (C20), use of natural resources (C21), and safety risk (C22) were all weighted and compared to one another. The pairwise comparison matrix of the criteria was made according to Eq. 1.

1 $$A=\left[\begin{array}{ccc}\begin{array}{c}\begin{array}{cc}{a}_{11}& \dots \end{array}\\ \begin{array}{cc}\vdots & \end{array}\end{array}& \begin{array}{c}\begin{array}{cc}{a}_{1j}& \dots \end{array}\\ \begin{array}{cc}\vdots & \end{array}\end{array}& \begin{array}{c}{a}_{1n}\\ \vdots \end{array}\\ \begin{array}{c}\begin{array}{cc}{a}_{i1}& \dots \end{array}\\ \begin{array}{cc}\vdots & \end{array}\end{array}& \begin{array}{c}\begin{array}{cc}{a}_{ij}& \dots \end{array}\\ \begin{array}{cc}\vdots & \end{array}\end{array}& \begin{array}{c}{a}_{in}\\ \vdots \end{array}\\ \begin{array}{cc}{a}_{1n}& \dots \end{array}& \begin{array}{cc}{a}_{nj}& \dots \end{array}& {a}_{nn}\end{array}\right],{a}_{ij}=1, {a}_{ij}=1/{a}_{ji} , {a}_{ji}\ne 0$$

Here, the criteria are ${a}_{1}$, ${a}_{1}$, …, ${a}_{n}$. “*n*” represents the total number of criteria. The relative importance of the two criteria was determined using a scale ranging from 1 to 9. In this case, 1 point means “low importance,” and 9 points “definitely more important.” The comparative weights are derived by finding the eigenvector (*ω*) with the respective ${\lambda }_{{\text{max}}}$ that satisfies ${A}_{w}$ = ${\lambda }_{{\text{max}}}$ *w*, where ${\lambda }_{{\text{max}}}$ is the largest eigenvalue of the matrix A (Ozturk 2018). Here, for the condition (–*A* − ${\lambda }_{{\text{max}}}$) *w* = 0, the corresponding ${\lambda }_{{\text{max}}}$ and the eigenvector *w* are found. Consistency index (CI) and consistency ratio (CR) are calculated to ensure consistency of subjective perception and accuracy of comparative weights (Eqs. 2 and 3).

2 $${\text{CI}}=({\lambda }_{{\text{max}}}-n)/(n-1)$$

where *n* is the number of criteria. The CR can be calculated as:

3 $${\text{CR}}={\text{CI}}/{\text{RI}}$$

The RI is determined for different-sized matrices, and its value is 1.64 for 22 × 22 matrices. The CR value should be below 0.1 for a reliable result.

## Results and discussion

AHP was used to assess different treatments for the removal of 2-MIB and geosmin from water sources. The assessment criteria and criteria weights for the decision-making process were chosen using AHP. Treatment options were classified according to AHP, and decision matrices were made. The treatment options for each approach were then ranked in order of importance. The analytical steps, results, and assessments are detailed in the subheadings below.

### Criteria weighting

On the basis of expert judgments and assessments found in the literature, various evaluation criteria were first identified (Table 2). The relative importance of the assessment criteria is used to define the three levels of the decision-making hierarchy. The first stage of the AHP technique was designed to find the optimal treatment options for removing 2-MIB and geosmin from sources of drinking water. The selection of assessment criteria is the second stage of AHP. Treatment choices are provided at the top level. The decision hierarchy is established, and then choices are assessed in pairs. The significance of the criterion was also evaluated in relation to the AHP. Distance performance, dependability, durability, vital operational consumables, and metabolite formation are more significant considerations, but complexity, ease of building, and equipment life are less crucial. The 22 assessment criteria’s weights were calculated using the AHP. The assessment criteria used in the decision-making process should have the intent of influencing the decision-making process. In other words, it is incorrect to assess merely on the basis of clearance or to accord equal weight to all evaluation criteria in order to get the most accurate findings when choosing the appropriate treatment plan.

**Table 2:** Evaluation of treatment options based on criteria

	A1	A2	A3	A4	A5	A6
(C1)	20–95% (∼60)/—< 40%	60–99% (∼90)/40–99% (∼85)	< 10–79% (∼65)/30–82 (∼72)	30–99% (∼85)/50–99% (∼90)	80–99% (∼90)/80–99% (∼90)	96–99% (∼98)/97–99% (∼98)
(C2)	Somewhat strong and dependable	Somewhat strong and dependable	Somewhat strong and dependable	Somewhat strong and dependable	Very dependable/durable. A successful record	Somewhat strong and dependable
(C3)	Somewhat difficult	Basic	Somewhat difficult	Somewhat difficult	Somewhat difficult	Somewhat difficult
(C4)	Low upkeep (≤ 2 weeks/year)	Modest upkeep (2–4 weeks/year)	Modest upkeep (2–4 weeks/year)	Modest upkeep (2–4 weeks/year)	Modest upkeep (2–4 weeks/year)	Modest upkeep (2–4 weeks/year)
(C5)	Manual and partially automated functioning	Manual and partially automated functioning	Complete automation with minimum manual intervention	Complete automation with minimum manual intervention	Fully automatic operation with minimal manual periodic	Manual and partially automated functioning
(C6)	Traditional building methods or an off-site packaging facility	Traditional building methods or an off-site packaging facility	Moderate amount of building activity or needs	Moderate amount of building activity or needs	Traditional building methods or an off-site packaging facility	Moderate level of construction requirements/activities
(C7)	3–5 main chemicals altogether	< 3 main chemicals altogether	3–5 main chemicals altogether	3–5 main chemicals altogether	3–5 main chemicals altogether	3–5 main chemicals altogether
(C8)	Rarely (5 years of complete replacement/supply)	Moderate (complete replacement or supply for 1 to 5 years)	Moderate (complete replacement or supply for 1 to 5 years)	Moderate (complete replacement or supply for 1 to 5 years)	Rarely (5 years of complete replacement/supply)	Moderate (complete replacement or supply for 1 to 5 years)
(C9)	50 installations and more than 10 years of expertise	50 installations and more than 10 years of expertise	10–50 deployments over 5–10	10–50 deployments over 5–10	50 installations and more than 10 years of expertise	10–50 deployments over 5–10
(C10)	2–4 waste streams with average levels of pollutants	There are < 2 waste streams with a modest pollution burden	There are < 2 waste streams with a modest pollution burden	There are < 2 waste streams with a modest pollution burden	There are < 2 waste streams with a modest pollution burden	2–4 waste streams with average levels of pollutants
(C11)	Alternatives to disposal are available at reasonable rates	Alternatives to disposal are available at reasonable rates	No, few, or simple disposal of waste streams	No, few, or simple disposal of waste streams	Alternatives to disposal are available at reasonable rates	Alternatives to disposal are available at reasonable rates
(C12)	70–90% average water production	70–90% average water production	Average water production > 90%	Average water production > 90%	Average water production > 90%	70–90% average water production
(C13)	Somewhat reliant on the quality of the influent water	Somewhat reliant on the quality of the influent water	Somewhat reliant on the quality of the influent water	Somewhat reliant on the quality of the influent water	Regardless of influence quality (expect extreme conditions)	Somewhat reliant on the quality of the influent water
(C14)	> 20 years	10–20 years	10–20 years	10–20 years	10–20 years	10–20 years
(C15)	Wide availability and extensive design expertise	Wide availability and extensive design expertise	Wide availability and extensive design expertise	Wide availability and extensive design expertise	Wide availability and extensive design expertise	Wide availability and extensive design expertise
(C16)	Largely unaffected by significant preprocessing needs	Pretreatment level is moderately necessary	High level of pretreatment necessary	High level of pretreatment necessary	High level of pretreatment necessary	Pretreatment level is moderately necessary
(C17)	By product formation unknown, low risk	By product formation unknown, low risk	Concerns about byproduct production may exist (limited info available)	Concerns about byproduct production may exist (limited info available)	By product formation unknown, low risk	Concerns about byproduct production may exist (limited info available)
(C18)	No restrictions	No restrictions, but little local expertise	No restrictions, but little local expertise	No restrictions, but little local expertise	No restrictions, but little local expertise	No restrictions, but little local expertise
(C19)	Need further treatment	No requires more treatment	Need further treatment	No requires more treatment	No requires more treatment	No requires more treatment
(C20)	A reasonable influence on the environment	A reasonable influence on the environment	Negative impacts on the environment	Negative impacts on the environment	A reasonable influence on the environment	A reasonable influence on the environment
(C21)	Not possible	Possible	Not possible	Not possible	Not possible	Not possible
(C22)	No risk	No risk	May pose a risk	May pose a risk	May pose a risk	No risk
	Li et al. (2019); Kim and Park (2021)	Beniwal et al. (2018); Yuan et al. (2020); Bong et al. (2021); Pan et al. (2016); Yu et al. (2016); Summers et al. (2013); Zoschke et al. (2011); Matsui et al. (2013); Kim et al. (2014); Yu et al. (2007)	Bu et al. (2017); Liu et al. (2017); Li et al. (2018)	Park et al. (2017a, b); Yao et al. (2017); Li et al. (2018); Ma et al. (2018); Ma et al. (2018); Yuan et al. (2018); Yaparatne et al. (2018); Visentin et al. (2019); Berlt et al. (2020); Huang et al. (2022); Soyluoglu et al. (2022); Bang et al. (2016); Kim et al. 2016); Kutschera et al. (2009); Mizuno et al. (2011); Yuan et al. (2013); Xie et al. (2015); Song and O’Shea (2007)	Doederer et al. (2019)	Jiang et al. (2022)

(C1): removal performance; (C2): reliability and durability; (C3): simplicity; (C4): ease of planned maintenance; (C5): ease of operation and routine maintenance; (C6): ease of construction; (C7): usage of various chemical; (C8): major operational consumables; (C9): proven/establishment technology; (C10): generated wastes; (C11): treatment/management requirements of wastes; (C12): water efficiency; (C13): security of supply; (C14): asset life; (C15): availability of technology; (C16): pretreatment requirements; (C17): by product/metabolite formation; (C18): suitability of application; (C19): additional treatments; (C20): environmental impacts; (C21): use of natural resources; (C22): safety risk. (A1): conventional treatments; (A2): adsorption oxidation; (A3): advanced oxidation; (A4): membrane filtration; (A5): hybrid processes

### Configuration of a binary comparison matrix

The pairwise comparison matrix of 22 criteria evaluated within the scope of the study is shown in Table 2. The pairwise comparison matrix was set up according to Eq. 1. This matrix was created to help compare any criteria with other criteria.

- In the first line, the “C1” criterion is compared with the other criteria. The most significant comparison of the “C1” criterion was formed by the “C4”–“C6” and “C21” criteria. “C1” criterion represents “Removal of 2-MIB and Geosmin,” “C4” criterion is “Ease of Planned Maintenance,” “C6” criterion is “Ease of Construction,” and “C21” criterion is “Use of Natural Resources.” Criterion “C1” was characterized as “Very Important” compared to the three mentioned criteria. Since the performance of treatment plants is determined by the removal rate of the targeted pollutant (Arvaniti et al. 2022), the “C1” criterion is “Very Important” compared to other criteria
- Criterion “C2” shown in line 2 represents “Reliability and Durability.” According to expert opinions, the “C2” criterion could not be scored as “9” or “1/9” according to any of the other criteria. The “C3” criterion, shown in line 3, represents “Simplicity”
- The “C3” criterion has almost the same degree of importance as all other criteria
- Criterion “C4” shown in line 4 represents “Ease of Planned Maintenance.” When the “C4” criterion is compared with the “C9”, “C14”, “C15”, “C18”, and “C22” criteria, it is emphasized that other criteria are “Very Important” by scoring “1/7”. The “C9” criterion represents “Proven/Establishment Technology.” “C14” criterion is the “Asset Life,” “C15” criterion is the “Availability of Technology,” “C18” criterion is the “Suitability of Application,” and “C22” criterion is the “Safety Risk” criterion. In treatment processes, the simplicity of any process is not always the first choice. However, proven technology in treatment processes, asset life, applicability of technology, availability of application, and safety risks is the first considerations in treatment processes (Ozturk 2018)
- In line 5, “C5” represents “Ease of Operation and Routine Maintenance.” The “C5” criterion has a similar degree of importance with the other criteria to which it is compared
- In line 6, the “C6” criterion represents “Ease of Construction.” The “C6” criterion was scored as “Very Important” as “1/7” when compared to the “C9”, “C14”, “C15”, “C18”, and “C22” criteria. “C6” criterion is very similar to the “C3” criterion, so close scores are given with the other criteria
- In line 7, the “C7” criterion represents the “Usage of Various Chemical,” and the 8th line “C8” represents the “Major Operational Consumables.” “C7” and “C8” criteria have similar importance levels with other criteria
- The “C9” criterion represents a “Proven/Establishment Technology.” “C9” criterion showed the most important match with “C21” criterion and got “7” points. The “C21” criterion represents the “Use of Natural Resources.” The use of natural resources in treatment processes is widely used in the adsorption process, but high performance has not been recorded in terms of 2-MIB and geosmin removal (Kim and Park 2021)
- “C10” criterion represents “Generated Wastes,” “C11” criterion represents “Treatment/Management Requirements of Wastes,” “C12” criterion represents “Water Efficiency,” and “C13” criterion represents “Security of Supply.” There were no significant differences between the aforementioned “C10”, “C11”, “C12”, and “C13” criteria compared to other criteria and their importance levels
- The “C14” criterion represents the “Asset Life,” the “C15” criterion represents the “Availability of Technology,” and the “C18” criterion represents the “Suitability of Application.” The most significant comparison of “C14” criterion, “C15” criterion, and “C18” criterion was determined by “C21”. It has been evaluated by experts that the asset life of a treatment process, the applicability of the treatment technology, or the application suitability criteria are very important compared to the natural resource use criteria
- “C16” criterion represents “Pretreatment Requirements,” and “C17” criterion represents “By Product/Metabolite Formation.” In the evaluation made by the experts, the difference between the criteria in which the “C16” and “C17” criteria are compared, and the degree of importance is quite close
- “C19” represents “Additional Treatment,” and “C20” represents “Environmental Impacts.” “C19” and “C20” criteria have close importance levels with other criteria
- The “C21” criterion represents the “Use of Natural Resources.” In the pairwise comparison matrix, the “C21” criterion is compared only with the “C22” criterion. The “C22” criterion is “Safety Risk.” In treatment processes, the safety risk of a process is defined as very important by giving “7” points compared to the use of natural resources

In the pairwise comparison matrix, the upper side of the “1” diagonal is filled based on the studies done by the experts. By taking the symmetry of the upper part of the “1” diagonal, the lower part of the diagonal was filled. After the pairwise comparison matrix was completed, each column was summed up within itself and their totals were written on the bottom line. In the continuation of the process, the cells were divided by the sum of the column to which they were connected, and the normalization process was completed. The normalization table is not included in the study. The statistical significance of the pairwise comparison matrix was determined according to Eq. 2 and Eq. 3. The “CI” value of the pairwise comparison matrix is 0.01. The “RI” value was accepted as 1.64 in 22 × 22 matrix (Alonso and Lamata 2006). For the pairwise comparison matrix to be statistically consistent, the “CR” value must be less than 0.1. The study is statistically significant as the CR value was calculated as 0.008 (Table 3).

**Table 3:**
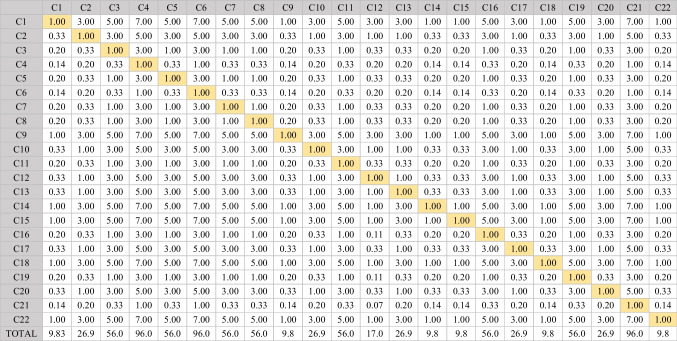
A comparison matrix of criteria

(C1): removal performance; (C2): reliability and durability; (C3): simplicity; (C4): ease of planned maintenance; (C5): ease of operation and routine maintenance; (C6): ease of construction; (C7): usage of various chemical; (C8): major operational consumables; (C9): proven/establishment technology; (C10): generated wastes; (C11): treatment/management requirements of wastes; (C12): water efficiency; (C13): security of supply; (C14): asset life; (C15): availability of technology; (C16): pretreatment requirements; (C17): by product/metabolite formation; (C18): suitability of application; (C19): additional treatments; (C20): environmental impacts; (C21): use of natural resources; (C22): safety risk

### Criteria weights

**Fig. 2:**
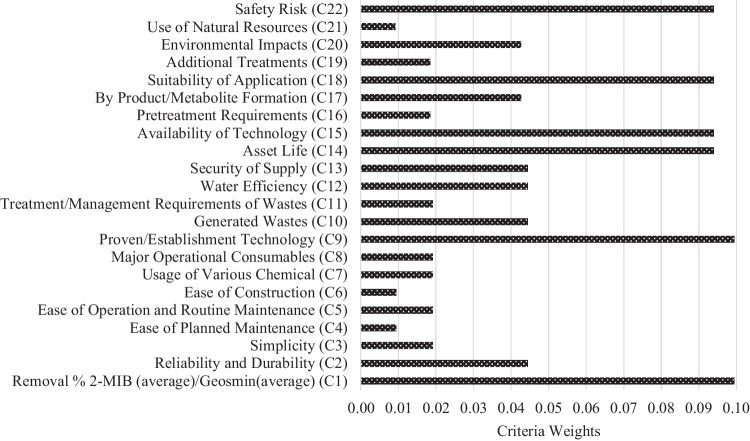
The weighted values of the criteria

AHP application starts with problem definition. In the second stage, after the hierarchical structure is created, the pairwise comparison matrix is created. After the pairwise comparison matrices are normalized, the priority vector is calculated. After the CR is found to be statistically significant (Eq. 3), a pairwise comparison matrix is created for the alternatives and the priority vector of the alternatives is calculated. The weighted values of each criterion are calculated. The weighted values of the criteria were calculated by taking the average of the values obtained in the normalization process. The weights of the criteria will be a determining factor in the ranking of the alternatives in the next stages. The weighted values of the criteria are shown in Fig. 2.

Weighted criteria can be divided into five groups according to their scores. In the first group, there are C1 and C9 criteria with 0.10 points. While the studies mostly focus on 2-MIB and geosmin removal, supporting the studies with proven technologies increases the reliability of the studies. Therefore, the fact that any 2-MIB and geosmin removal studies have been performed with proven treatment processes encourages researchers to put it into practice. The second group of weighted criteria includes C14, C15, C18, and C22 criteria with 0.09 points. The criteria in the second group are the longevity of the treatment processes, the usability of the technology, the suitability of the application, and the safety risks. The criteria in this group generally cover the design of treatment processes, the existence and sustainability of the technologies used, and their safety. The criteria of C2, C10, C12, C13, C17, and C20 in the third group got 0.04 points. The criteria in this group are water efficiency, waste generation, and sustainability of treatment processes. The third group criteria are the second most clustered group among all criteria. This is because this group is related to the general operation of treatment processes. All criteria in this group are valuable in terms of the environmental effects of treatment processes. The fourth group of criteria includes C3, C5, C7, C8, C11, C16, and C19. The criteria in the fourth group deserved 0.02 points. The criteria in the fourth group are the part where the most clustering is seen. The criteria in this group cover the entire flow chart of a treatment process. In this context, chemicals used, additional treatment requirements, and technical maintenance requirements are included in the criteria. C4, C6, and C21 criteria in the fifth group received 0.01 points. This group provided the least added value in ranking the alternatives. Criteria in this group require specific installation or use of materials. Therefore, the validity of these criteria for all treatment processes is debatable.

### Ranking of alternatives

In ordering the alternatives, a pairwise comparison of all treatment processes was made on the basis of criteria. In the process of pairwise comparison of alternatives, the same methodology was used as the weighting of the criteria. The methodology applied while listing the alternatives is given in the appendices. The score to be used in ranking each alternative was calculated by multiplying the weighted score of the criteria and the average of the score of the relevant alternative relative to the other alternatives. The order of treatment processes selected as alternatives is given in Table 4.

(A1): Zamyadi et al. (2015) emphasized in their research introduction part, removal efficiency of conventional chemical-physical (coagulation-flocculation, clarification, and filtration) and disinfection treatment processes for the removal of extracellular geosmin and MIB is very low, at less than 20%. Similar to other studies, Kim et al. 2016) stated that conventional water treatment processes (sedimentation, chlorination, coagulation, and flocculation) cannot remove geosmin and 2-MIB efficiently. This has not changed in the publications completed in recent years, and in the studies of Cerón-Vivas et al. (2023), it was re-emphasized that traditional treatment processes in water treatment are ineffective in removing 2-MIB/GSM.

(A2): The adsorption process is one of the most common cleaning processes in drinking water and wastewater treatment (Yousef et al. 2020). Activated carbon (GAC/PAC) as well as natural, novel, and biosorbents are used as adsorbents in the adsorption process (Saleem et al. 2019). The main advantages of removing 2-MIB and geosmin from the adsorption process can be listed as high removal efficiency, established/established technology, waste generated, treatment/management requirements of waste, water use efficiency, security of supply, and safety risk. The main concern in adsorption process applications is the important operating consumables. Further sand filtration treatment is required to completely remove septic tank odors. However, the adsorption method using PAC and GAC after conventional treatment could remove 2-MIB and geosmin more effectively (Drikas et al. 2009). Adsorption on activated carbon offers significant advantages over other treatment methods, such as lower energy consumption and no by-product formation (Bui et al. 2016). The main benefits of removing 2-MIB and geosmin from the oxidation process can be listed as moderate removal efficiency, ease of operation and routine maintenance, waste generated, water use efficiency, and waste disposal/management requirements. The main issues in the application of adsorption processes are significant operating consumables, safety hazards, additional treatment, and by-product/metabolite formation.

(A3): Advanced oxidation processes (AOP) are broadly defined as a group of chemical treatment processes aimed at the oxidative removal of organic (and sometimes inorganic) substances from water and wastewater by reaction with hydroxyl radicals (·OH). Although AOPs are an effective method for removing many organic pollutants, their main limitation compared to other conventional treatments is cost (Sarria et al. 2003). The advantages of advanced oxidation processes in 2-MIB and geosmin removal include high removal efficiency, low waste generation, high water efficiency, technical availability, and applicability (Yuan et al. 2013). Critical operating consumables, use of various chemically validated/proven technologies, need for additional treatment, waste disposal/management requirements, security of supply, and pretreatment requirements are the main issues in applying advanced oxidation processes (Brinkmann et al. 2016). The advanced oxidation process is divided into chemical process, photochemical process, and electrochemical process. Electrochemical processes such as electro-oxidation, electro-Fenton, or electro-peroxyketone may not be suitable for water treatment plants in today’s conditions due to the high cost of electricity (Ghernaout et al. 2020). However, it will become increasingly important for wastewater treatment plants in the coming years. UV-based photochemical further oxidation processes are not recommended due to the high demand for pretreatment and the fact that the technology has not yet established itself in wastewater treatment plants. Chemical advanced oxidation processes such as ozone, ozone-peroxide, Fenton’s process, and ultrasonic treatment are particularly common treatments for deodorization and deodorization (Dębowski et al. 2022). Today, the pilot-scale ozone advanced oxidation process can be successfully applied for the removal of 2-MIB and geosmin in some wastewater treatment plants (Park et al. 2017a, b). The biggest problem with using ozone is the formation of bromate (Gounden and Jonnalagadda 2019). By lowering the pH of the water below 7, the bromate problem was also addressed (Jahan et al. 2021).

(A4): Membrane filtration processes (microfiltration (MF), ultrafiltration (UF), nanofiltration (NF), and reverse osmosis (RO)) are increasingly used treatment technologies in water and wastewater treatment (ElSherbiny and Panglisch 2021). Membrane filtration has high removal efficiency in removing 2-MIB and geosmin as well as a large number of actinomycetes (Kim et al. 2016). Advantages of membrane filtration process in 2-MIB and geosmin removal include high removal efficiency, easy planned maintenance and operation, reliability and longevity, mature/mature technology, waste disposal/management requirements, and security of supply (Othman et al. 2022). The main problem when applying the membrane filtration process is routine maintenance.

(A5): Hybrid processes consist of a combination of several treatment techniques involving different removal mechanisms. Hybrid processes have been used to remove various pollutants from drinking water and wastewater. The hybrid process can also be used to modernize existing conventional wastewater treatment plants to remove 2-MIB and geosmin. The main disadvantage of the mixing process is that it is rarely applied on a large scale. Many treatment combinations such as adsorption/membrane filtration and membrane filtration/advanced oxidation can be evaluated after conventional water treatment especially in 2-MIB and geosmin removal.

## Sensitivity analysis

Sensitivity analysis studies were taken from the article published by Srdjevic et al. (2012). The ultimate prioritization of alternatives is profoundly influenced by the assigned weights to the criteria. Even minor adjustments in these weights can lead to substantial shifts in the final ranking. As the criteria weights stem from the subjective assessment of the decision maker, it becomes imperative to explore how alterations in criteria scores or assigned weights might impact the ranking of alternatives. Clearly, sensitivity analysis serves as a valuable tool, offering insights into the stability of the solution—specifically, the ranking of alternatives and the identification of the optimal choice in a multicriterial context.

### Procedure for performing the sensitivity analysis

The sensitivity analysis of the solution described in the previous section is performed following the strategy to increase the weight of the criteria: removal % 2-MIB (average)/geosmin (average) (C1), proven/establishment technology (C9), asset life (C14), availability of technology (C15), suitability of application (C18), and safety risk (C22). The implementation of the removal of % 2-MIB/geosmin from water treatment plants by proven/establishment technology will be prioritized by managers, especially due to economic concerns. On the other hand, asset life, availability of technology, suitability of application, and safety risk are becoming important issues especially with the industrial sector facing a global economic crisis.

Through increasing or decreasing the weights of the highest scoring criteria, removal % 2-MIB/geosmin, proven/establishment technology, asset life, availability of technology, suitability of application, safety risk, resulting changes in the priorities, and ranking of the alternatives can be observed. If the ranking is highly sensitive to small changes in the related criteria weights, a careful review of the weights would be recommended as the final outcome of the sensitivity analysis.

When the value of the selected criterion (removal % 2-MIB /geosmin, proven/establishment technology, asset life, availability of technology, suitability of application, and safety risk in this example) is varied from 0 to 1, and the weights of the other criteria are recalculated in a way that the ratios between them are kept constant (Erkut and Tarimcilar 1991).

Suppose that we first want to vary the weight of the criterion removal % 2-MIB /geosmin, ${w}_{1}$. As noted in the previous section, the sum of all criteria weights is one

4 $${w}_{1}+{w}_{2}+{w}_{3}+\dots +{w}_{20}+{w}_{21}+{w}_{22}=1$$

Defining ${p}_{1}={w}_{2}/{w}_{3}$, ${p}_{2}={w}_{4}/{w}_{3}$, ${p}_{3}={w}_{5}/{w}_{3}$, …, ${p}_{18}={w}_{20}/{w}_{3}$, ${p}_{19}={w}_{21}/{w}_{3}$, ${p}_{20}={w}_{22}/{w}_{3}$, and inserting ${p}_{1}-{p}_{20}$ into Eq. 4, we obtain

5 $${w}_{1}+{p}_{1}{w}_{3}+{w}_{3}+{p}_{2}{w}_{3}+{p}_{3}{w}_{3}+\dots +{p}_{18}{w}_{3}+{p}_{19}{w}_{3}+{p}_{20}{w}_{3}=1$$

and ${w}_{3}$ as a function of ${w}_{1}$

6 $${w}_{3}=\frac{1-{w}_{1}}{1+{p}_{1}+{p}_{2}+{p}_{3}+{p}_{3}+\dots +{p}_{15}+{p}_{16}+{p}_{17}+{p}_{18}+{p}_{19}+{p}_{20}}$$

which implies that all other criteria weights are also functions of a single variable *w*1:

7 $$\begin{array}{c}{w}_{2}=\frac{{p}_{1}(1-{w}_{1})}{1+{p}_{1}+{p}_{2}+{p}_{3}+{p}_{3}+\dots +{p}_{15}+{p}_{16}+{p}_{17}+{p}_{18}+{p}_{19}+{p}_{20}}\\ \begin{array}{c}{w}_{4}=\frac{{p}_{2}(1-{w}_{1})}{1+{p}_{1}+{p}_{2}+{p}_{3}+{p}_{3}+\dots +{p}_{15}+{p}_{16}+{p}_{17}+{p}_{18}+{p}_{19}+{p}_{20}}\\ {w}_{5}=\frac{{p}_{3}(1-{w}_{1})}{1+{p}_{1}+{p}_{2}+{p}_{3}+{p}_{3}+\dots +{p}_{15}+{p}_{16}+{p}_{17}+{p}_{18}+{p}_{19}+{p}_{20}}\\ {w}_{19}=\frac{{p}_{21}(1-{w}_{1})}{1+{p}_{1}+{p}_{2}+{p}_{3}+{p}_{3}+\dots +{p}_{15}+{p}_{16}+{p}_{17}+{p}_{18}+{p}_{19}+{p}_{20}}\end{array}\\ {w}_{20}=\frac{{p}_{22}(1-{w}_{1})}{1+{p}_{1}+{p}_{2}+{p}_{3}+{p}_{3}+\dots +{p}_{15}+{p}_{16}+{p}_{17}+{p}_{18}+{p}_{19}+{p}_{20}}\end{array}$$

For the criteria weights obtained in the previous section (${w}_{2}$ = 0.045, ${w}_{3}$ = 0.019, ${w}_{4}$ = 0.010, ${w}_{5}$ = 0.019, ${w}_{6}$ = 0.010, ${w}_{7}$= 0.019, ${w}_{8}$ = 0.019, ${w}_{9}$ = 0.099, ${w}_{10}$ = 0.045, ${w}_{11}$ = 0.019, ${w}_{12}$ = 0.045, ${w}_{13}$ = 0.045, ${w}_{14}$ = 0.094, ${w}_{15}$ = 0.094, ${w}_{16}$= 0.019, ${w}_{17}$= 0.043, ${w}_{18}$= 0.094, ${w}_{19}$= 0.019, ${w}_{20}$= 0.043, ${w}_{21}$ = 0.009, and ${w}_{22}$ = 0.094), the values of ${p}_{1}+{p}_{20}$ are as follows: ${p}_{1}$ = 2.319, ${p}_{2}$ = 0.495, ${p}_{3}$ = 1.000, ${p}_{4}$ = 0.495, ${p}_{5}$ = 1.000, ${p}_{6}$ = 1.000, ${p}_{7}$ = 5.180, ${p}_{8}$ = 2.319, ${p}_{9}$ = 1.000, ${p}_{10}$ = 2.319, ${p}_{11}$ = 2.319, ${p}_{12}$ = 4.901, ${p}_{13}$ = 4.901, ${p}_{14}$ = 0.969, ${p}_{15}$ = 2.226, ${p}_{16}$ = 4.901, ${p}_{17}$ = 0.969, ${p}_{18}$ = 2.226, ${p}_{19}$ = 0.476, and ${p}_{20}$ = 4.901.

When ${w}_{1}$ is set, the weights of the other criteria are calculated using Eqs. 6 and 7. The results of the sensitivity analysis indicate that the rankings of the criteria contributing the most and the least to the ranking of alternatives in the AHP have not changed. This signifies that the conducted study is meaningful and applicable in terms of sensitivity analysis.

## Conclusion

This study is to determine the order of importance of different treatment processes, which are especially focused on the removal of T&O components, with the analytical hierarchy process, which is a multi-criteria decision-making method. The most studied compounds are GSM and MIB, as they have been identified as the major T&O-causing compounds in drinking water. MIB has been found to be more difficult to troubleshoot than GSM. This is the first study using 2-MIB and AHP methodology for removal modification in water treatment plants. The existing literature reviewed here has brought up various treatment processes that have come to the fore especially in recent years for the removal of T&O compounds from the aquatic environment. First, the treatment processes examined in this study were prioritized according to 22 different new criteria. Among the criteria examined, it was determined that the most significant criteria in prioritizing the treatment processes were the removal efficiency and proven technology with 0.1 points. Second, the weight scores of the treatment processes used in the study were calculated for each criterion. Comparison of the performances of different treatment processes was also made. Ozone-based AOPs appear to be particularly promising technologies for T&O treatment of the aquatic environment, offering rapid kinetics, high degree of mineralization, and performance advantages. In this study, the optimal treatment option for removing 2-MIB and geosmin from water sources was chosen using analytical decision-making techniques like AHP. AHP was used to develop the assessment criteria and weight them in the study of the treatment options. The AOPs for 2-MIB and geosmin are the best treatment options. The collected results from the decision procedures can be totaled and ordered from low to high if a precise alternative ranking including the outcomes of all approaches is needed. This method led to the conclusion that advanced oxidation processes had the highest priority, followed by membrane filtration, adsorption, oxidation, hybrid processes, and traditional treatment procedures.

Instead of the AHP, several other multi-criteria decision making (MCDM) methods can be used to determine the most appropriate process for a drinking water treatment facility. TOPSIS (Technique for Order Preference by Similarity to Ideal Solution): TOPSIS is a method that focuses on finding the alternative that is the closest to the ideal solution and farthest from the negative ideal solution. It considers both the positive and negative aspects of the criteria (Yahya et al. 2020). PROMETHEE (Preference Ranking Organization METHod for Enrichment Evaluations): PROMETHEE is a family of decision-making methods that compare alternatives based on partial pre-orders. It provides a preference ranking for the alternatives (Gichamo et al. 2020). Vikor Method (Vikor): The Vikor method is used to select the best alternative from a set of alternatives by considering both the maximum group utility and the minimum individual regret (Dursun 2016). When choosing a particular MCDM method, the nature of your decision problem, the preferences of the decision makers, the availability of data, and the complexity of the decision-making process should be considered. Each method has its strengths and weaknesses, and which one is most appropriate depends on the specific characteristics of the problem at hand.

### Supplementary Information

ESM 1 (DOCX 107 kb)

## Data Availability

The datasets utilized and/or investigated during the current research are available from the corresponding author upon reasonable request.

## Code availability

All software used in the present study was used with the license of each code.
