# Supplementary material for: The analytic hierarchy process method to design applicable decision making for the effective removal of 2-MIB and geosmin in water sources
Source: Environ Sci Pollut Res Int. 2024 Jan 17;31(8):12431–45. doi: 10.1007/s11356-024-31848-7 (PMC10869403; doi:10.1007/s11356-024-31848-7)
Supplement: Supplementary file 1 — (DOCX 107 kb) [file 11356_2024_31848_MOESM1_ESM.docx]

**Appendix 1 Used questionnaire list for collecting expert opinions**

| Summary  Many researchers have reported that the formation of taste and odor components in water can cause major problems for the environment and human health. Due to global climate change, wastewater initiatives, aquaculture and agricultural activities, one of the main ways these pollutants enter the environment may be through the increase of organic structures in the aquatic environment. One of the routes of human exposure is through drinking water. Unfortunately, the drinking water treatment systems that exist today are not designed for the removal of taste and odor components, which unfortunately makes existing treatment systems unsuitable for the removal of taste and odor components. Therefore, it is necessary to modernize existing conventional equipment. However, which processing technologies will be used in this modernization process, how these processing technologies will be determined, and according to what standards, remain questions. In this study, 2-MIB and geosmin were selected as sample contaminants, and treatment alternatives were identified in the literature review. AHP is one of the multi-criteria decision-making methods used to determine the best treatment option. In this context, the aim was to incorporate expert opinion on water treatment into the study. The pairwise comparison matrix below should be constructed according to the criteria outlined in the 9-point scoring system. Based on the responses received, the criteria are weighted, and the alternatives are ranked against the criteria.  Thanks for your support, participation and sharing your valuable comments to this study.  Best regards... |
| --- |

**Table 5.** Pairwise comparison matrix used to compare criteria

|  | Extreme Importance | Very Strong Importance | Strong Importance | Moderate Importance | Equal Importance | Moderate Importance | Strong Importance | Very Strong Importance | Extreme Importance |  |
| --- | --- | --- | --- | --- | --- | --- | --- | --- | --- | --- |
| C1 | 9 | 7 | 5 | 3 | 1 | 3 | 5 | 7 | 9 | C1 |
| C1 | 9 | 7 | 5 | 3 | 1 | 3 | 5 | 7 | 9 | C2 |
| C1 | 9 | 7 | 5 | 3 | 1 | 3 | 5 | 7 | 9 | C3 |
| C1 | 9 | 7 | 5 | 3 | 1 | 3 | 5 | 7 | 9 | C4 |
| C1 | 9 | 7 | 5 | 3 | 1 | 3 | 5 | 7 | 9 | C5 |
| C1 | 9 | 7 | 5 | 3 | 1 | 3 | 5 | 7 | 9 | C6 |
| C1 | 9 | 7 | 5 | 3 | 1 | 3 | 5 | 7 | 9 | C7 |
| C1 | 9 | 7 | 5 | 3 | 1 | 3 | 5 | 7 | 9 | C8 |
| C1 | 9 | 7 | 5 | 3 | 1 | 3 | 5 | 7 | 9 | C9 |
| C1 | 9 | 7 | 5 | 3 | 1 | 3 | 5 | 7 | 9 | C10 |
| C1 | 9 | 7 | 5 | 3 | 1 | 3 | 5 | 7 | 9 | C11 |
| C1 | 9 | 7 | 5 | 3 | 1 | 3 | 5 | 7 | 9 | C12 |
| C1 | 9 | 7 | 5 | 3 | 1 | 3 | 5 | 7 | 9 | C13 |
| C1 | 9 | 7 | 5 | 3 | 1 | 3 | 5 | 7 | 9 | C14 |
| C1 | 9 | 7 | 5 | 3 | 1 | 3 | 5 | 7 | 9 | C15 |
| C1 | 9 | 7 | 5 | 3 | 1 | 3 | 5 | 7 | 9 | C16 |
| C1 | 9 | 7 | 5 | 3 | 1 | 3 | 5 | 7 | 9 | C17 |
| C1 | 9 | 7 | 5 | 3 | 1 | 3 | 5 | 7 | 9 | C18 |
| C1 | 9 | 7 | 5 | 3 | 1 | 3 | 5 | 7 | 9 | C19 |
| C1 | 9 | 7 | 5 | 3 | 1 | 3 | 5 | 7 | 9 | C20 |
| C1 | 9 | 7 | 5 | 3 | 1 | 3 | 5 | 7 | 9 | C21 |
| C2 | 9 | 7 | 5 | 3 | 1 | 3 | 5 | 7 | 9 | C22 |
| C2 | 9 | 7 | 5 | 3 | 1 | 3 | 5 | 7 | 9 | C2 |
| C2 | 9 | 7 | 5 | 3 | 1 | 3 | 5 | 7 | 9 | C3 |
| C2 | 9 | 7 | 5 | 3 | 1 | 3 | 5 | 7 | 9 | C4 |
| C2 | 9 | 7 | 5 | 3 | 1 | 3 | 5 | 7 | 9 | C5 |
| C2 | 9 | 7 | 5 | 3 | 1 | 3 | 5 | 7 | 9 | C6 |
| C2 | 9 | 7 | 5 | 3 | 1 | 3 | 5 | 7 | 9 | C7 |

**Table 5.** (continued)

| C2 | 9 | 7 | 5 | 3 | 1 | 3 | 5 | 7 | 9 | C8 |
| --- | --- | --- | --- | --- | --- | --- | --- | --- | --- | --- |
| C2 | 9 | 7 | 5 | 3 | 1 | 3 | 5 | 7 | 9 | C9 |
| C2 | 9 | 7 | 5 | 3 | 1 | 3 | 5 | 7 | 9 | C10 |
| C2 | 9 | 7 | 5 | 3 | 1 | 3 | 5 | 7 | 9 | C11 |
| C2 | 9 | 7 | 5 | 3 | 1 | 3 | 5 | 7 | 9 | C12 |
| C2 | 9 | 7 | 5 | 3 | 1 | 3 | 5 | 7 | 9 | C13 |
| C2 | 9 | 7 | 5 | 3 | 1 | 3 | 5 | 7 | 9 | C14 |
| C2 | 9 | 7 | 5 | 3 | 1 | 3 | 5 | 7 | 9 | C15 |
| C2 | 9 | 7 | 5 | 3 | 1 | 3 | 5 | 7 | 9 | C16 |
| C2 | 9 | 7 | 5 | 3 | 1 | 3 | 5 | 7 | 9 | C17 |
| C2 | 9 | 7 | 5 | 3 | 1 | 3 | 5 | 7 | 9 | C18 |
| C2 | 9 | 7 | 5 | 3 | 1 | 3 | 5 | 7 | 9 | C19 |
| C2 | 9 | 7 | 5 | 3 | 1 | 3 | 5 | 7 | 9 | C20 |
| C3 | 9 | 7 | 5 | 3 | 1 | 3 | 5 | 7 | 9 | C21 |
| C3 | 9 | 7 | 5 | 3 | 1 | 3 | 5 | 7 | 9 | C22 |
| C3 | 9 | 7 | 5 | 3 | 1 | 3 | 5 | 7 | 9 | C3 |
| C3 | 9 | 7 | 5 | 3 | 1 | 3 | 5 | 7 | 9 | C4 |
| C3 | 9 | 7 | 5 | 3 | 1 | 3 | 5 | 7 | 9 | C5 |
| C3 | 9 | 7 | 5 | 3 | 1 | 3 | 5 | 7 | 9 | C6 |
| C3 | 9 | 7 | 5 | 3 | 1 | 3 | 5 | 7 | 9 | C7 |
| C3 | 9 | 7 | 5 | 3 | 1 | 3 | 5 | 7 | 9 | C8 |
| C3 | 9 | 7 | 5 | 3 | 1 | 3 | 5 | 7 | 9 | C9 |
| C3 | 9 | 7 | 5 | 3 | 1 | 3 | 5 | 7 | 9 | C10 |
| C3 | 9 | 7 | 5 | 3 | 1 | 3 | 5 | 7 | 9 | C11 |
| C3 | 9 | 7 | 5 | 3 | 1 | 3 | 5 | 7 | 9 | C12 |
| C3 | 9 | 7 | 5 | 3 | 1 | 3 | 5 | 7 | 9 | C13 |
| C3 | 9 | 7 | 5 | 3 | 1 | 3 | 5 | 7 | 9 | C14 |
| C3 | 9 | 7 | 5 | 3 | 1 | 3 | 5 | 7 | 9 | C15 |
| C3 | 9 | 7 | 5 | 3 | 1 | 3 | 5 | 7 | 9 | C16 |
| C3 | 9 | 7 | 5 | 3 | 1 | 3 | 5 | 7 | 9 | C17 |
| C3 | 9 | 7 | 5 | 3 | 1 | 3 | 5 | 7 | 9 | C18 |
| C3 | 9 | 7 | 5 | 3 | 1 | 3 | 5 | 7 | 9 | C19 |
| C4 | 9 | 7 | 5 | 3 | 1 | 3 | 5 | 7 | 9 | C20 |
| C4 | 9 | 7 | 5 | 3 | 1 | 3 | 5 | 7 | 9 | C21 |
| C4 | 9 | 7 | 5 | 3 | 1 | 3 | 5 | 7 | 9 | C22 |
| C4 | 9 | 7 | 5 | 3 | 1 | 3 | 5 | 7 | 9 | C4 |
| C4 | 9 | 7 | 5 | 3 | 1 | 3 | 5 | 7 | 9 | C5 |
| C4 | 9 | 7 | 5 | 3 | 1 | 3 | 5 | 7 | 9 | C6 |
| C4 | 9 | 7 | 5 | 3 | 1 | 3 | 5 | 7 | 9 | C7 |
| C4 | 9 | 7 | 5 | 3 | 1 | 3 | 5 | 7 | 9 | C8 |
| C4 | 9 | 7 | 5 | 3 | 1 | 3 | 5 | 7 | 9 | C9 |
| C4 | 9 | 7 | 5 | 3 | 1 | 3 | 5 | 7 | 9 | C10 |
| C4 | 9 | 7 | 5 | 3 | 1 | 3 | 5 | 7 | 9 | C11 |
| C4 | 9 | 7 | 5 | 3 | 1 | 3 | 5 | 7 | 9 | C12 |
| C4 | 9 | 7 | 5 | 3 | 1 | 3 | 5 | 7 | 9 | C13 |
| C4 | 9 | 7 | 5 | 3 | 1 | 3 | 5 | 7 | 9 | C14 |
| C4 | 9 | 7 | 5 | 3 | 1 | 3 | 5 | 7 | 9 | C15 |
| C4 | 9 | 7 | 5 | 3 | 1 | 3 | 5 | 7 | 9 | C16 |
| C4 | 9 | 7 | 5 | 3 | 1 | 3 | 5 | 7 | 9 | C17 |
| C4 | 9 | 7 | 5 | 3 | 1 | 3 | 5 | 7 | 9 | C18 |
| C5 | 9 | 7 | 5 | 3 | 1 | 3 | 5 | 7 | 9 | C19 |
| C5 | 9 | 7 | 5 | 3 | 1 | 3 | 5 | 7 | 9 | C20 |
| C5 | 9 | 7 | 5 | 3 | 1 | 3 | 5 | 7 | 9 | C21 |
| C5 | 9 | 7 | 5 | 3 | 1 | 3 | 5 | 7 | 9 | C22 |
| C5 | 9 | 7 | 5 | 3 | 1 | 3 | 5 | 7 | 9 | C5 |
| C5 | 9 | 7 | 5 | 3 | 1 | 3 | 5 | 7 | 9 | C6 |

**Table 5.** (continued)

| C5 | 9 | 7 | 5 | 3 | 1 | 3 | 5 | 7 | 9 | C7 |
| --- | --- | --- | --- | --- | --- | --- | --- | --- | --- | --- |
| C5 | 9 | 7 | 5 | 3 | 1 | 3 | 5 | 7 | 9 | C8 |
| C5 | 9 | 7 | 5 | 3 | 1 | 3 | 5 | 7 | 9 | C9 |
| C5 | 9 | 7 | 5 | 3 | 1 | 3 | 5 | 7 | 9 | C10 |
| C5 | 9 | 7 | 5 | 3 | 1 | 3 | 5 | 7 | 9 | C11 |
| C5 | 9 | 7 | 5 | 3 | 1 | 3 | 5 | 7 | 9 | C12 |
| C5 | 9 | 7 | 5 | 3 | 1 | 3 | 5 | 7 | 9 | C13 |
| C5 | 9 | 7 | 5 | 3 | 1 | 3 | 5 | 7 | 9 | C14 |
| C5 | 9 | 7 | 5 | 3 | 1 | 3 | 5 | 7 | 9 | C15 |
| C5 | 9 | 7 | 5 | 3 | 1 | 3 | 5 | 7 | 9 | C16 |
| C5 | 9 | 7 | 5 | 3 | 1 | 3 | 5 | 7 | 9 | C17 |
| C6 | 9 | 7 | 5 | 3 | 1 | 3 | 5 | 7 | 9 | C18 |
| C6 | 9 | 7 | 5 | 3 | 1 | 3 | 5 | 7 | 9 | C19 |
| C6 | 9 | 7 | 5 | 3 | 1 | 3 | 5 | 7 | 9 | C20 |
| C6 | 9 | 7 | 5 | 3 | 1 | 3 | 5 | 7 | 9 | C21 |
| C6 | 9 | 7 | 5 | 3 | 1 | 3 | 5 | 7 | 9 | C22 |
| C6 | 9 | 7 | 5 | 3 | 1 | 3 | 5 | 7 | 9 | C6 |
| C6 | 9 | 7 | 5 | 3 | 1 | 3 | 5 | 7 | 9 | C7 |
| C6 | 9 | 7 | 5 | 3 | 1 | 3 | 5 | 7 | 9 | C8 |
| C6 | 9 | 7 | 5 | 3 | 1 | 3 | 5 | 7 | 9 | C9 |
| C6 | 9 | 7 | 5 | 3 | 1 | 3 | 5 | 7 | 9 | C10 |
| C6 | 9 | 7 | 5 | 3 | 1 | 3 | 5 | 7 | 9 | C11 |
| C6 | 9 | 7 | 5 | 3 | 1 | 3 | 5 | 7 | 9 | C12 |
| C6 | 9 | 7 | 5 | 3 | 1 | 3 | 5 | 7 | 9 | C13 |
| C6 | 9 | 7 | 5 | 3 | 1 | 3 | 5 | 7 | 9 | C14 |
| C6 | 9 | 7 | 5 | 3 | 1 | 3 | 5 | 7 | 9 | C15 |
| C6 | 9 | 7 | 5 | 3 | 1 | 3 | 5 | 7 | 9 | C16 |
| C7 | 9 | 7 | 5 | 3 | 1 | 3 | 5 | 7 | 9 | C17 |
| C7 | 9 | 7 | 5 | 3 | 1 | 3 | 5 | 7 | 9 | C18 |
| C7 | 9 | 7 | 5 | 3 | 1 | 3 | 5 | 7 | 9 | C19 |
| C7 | 9 | 7 | 5 | 3 | 1 | 3 | 5 | 7 | 9 | C20 |
| C7 | 9 | 7 | 5 | 3 | 1 | 3 | 5 | 7 | 9 | C21 |
| C7 | 9 | 7 | 5 | 3 | 1 | 3 | 5 | 7 | 9 | C22 |
| C7 | 9 | 7 | 5 | 3 | 1 | 3 | 5 | 7 | 9 | C7 |
| C7 | 9 | 7 | 5 | 3 | 1 | 3 | 5 | 7 | 9 | C8 |
| C7 | 9 | 7 | 5 | 3 | 1 | 3 | 5 | 7 | 9 | C9 |
| C7 | 9 | 7 | 5 | 3 | 1 | 3 | 5 | 7 | 9 | C10 |
| C7 | 9 | 7 | 5 | 3 | 1 | 3 | 5 | 7 | 9 | C11 |
| C7 | 9 | 7 | 5 | 3 | 1 | 3 | 5 | 7 | 9 | C12 |
| C7 | 9 | 7 | 5 | 3 | 1 | 3 | 5 | 7 | 9 | C13 |
| C7 | 9 | 7 | 5 | 3 | 1 | 3 | 5 | 7 | 9 | C14 |
| C7 | 9 | 7 | 5 | 3 | 1 | 3 | 5 | 7 | 9 | C15 |
| C8 | 9 | 7 | 5 | 3 | 1 | 3 | 5 | 7 | 9 | C16 |
| C8 | 9 | 7 | 5 | 3 | 1 | 3 | 5 | 7 | 9 | C17 |
| C8 | 9 | 7 | 5 | 3 | 1 | 3 | 5 | 7 | 9 | C18 |
| C8 | 9 | 7 | 5 | 3 | 1 | 3 | 5 | 7 | 9 | C19 |
| C8 | 9 | 7 | 5 | 3 | 1 | 3 | 5 | 7 | 9 | C20 |
| C8 | 9 | 7 | 5 | 3 | 1 | 3 | 5 | 7 | 9 | C21 |
| C8 | 9 | 7 | 5 | 3 | 1 | 3 | 5 | 7 | 9 | C22 |
| C8 | 9 | 7 | 5 | 3 | 1 | 3 | 5 | 7 | 9 | C8 |
| C8 | 9 | 7 | 5 | 3 | 1 | 3 | 5 | 7 | 9 | C9 |
| C8 | 9 | 7 | 5 | 3 | 1 | 3 | 5 | 7 | 9 | C10 |
| C8 | 9 | 7 | 5 | 3 | 1 | 3 | 5 | 7 | 9 | C11 |
| C8 | 9 | 7 | 5 | 3 | 1 | 3 | 5 | 7 | 9 | C12 |
| C8 | 9 | 7 | 5 | 3 | 1 | 3 | 5 | 7 | 9 | C13 |
| C8 | 9 | 7 | 5 | 3 | 1 | 3 | 5 | 7 | 9 | C14 |

**Table 5.** (continued)

| C9 | 9 | 7 | 5 | 3 | 1 | 3 | 5 | 7 | 9 | C15 |
| --- | --- | --- | --- | --- | --- | --- | --- | --- | --- | --- |
| C9 | 9 | 7 | 5 | 3 | 1 | 3 | 5 | 7 | 9 | C16 |
| C9 | 9 | 7 | 5 | 3 | 1 | 3 | 5 | 7 | 9 | C17 |
| C9 | 9 | 7 | 5 | 3 | 1 | 3 | 5 | 7 | 9 | C18 |
| C9 | 9 | 7 | 5 | 3 | 1 | 3 | 5 | 7 | 9 | C19 |
| C9 | 9 | 7 | 5 | 3 | 1 | 3 | 5 | 7 | 9 | C20 |
| C9 | 9 | 7 | 5 | 3 | 1 | 3 | 5 | 7 | 9 | C21 |
| C9 | 9 | 7 | 5 | 3 | 1 | 3 | 5 | 7 | 9 | C22 |
| C9 | 9 | 7 | 5 | 3 | 1 | 3 | 5 | 7 | 9 | C9 |
| C9 | 9 | 7 | 5 | 3 | 1 | 3 | 5 | 7 | 9 | C10 |
| C9 | 9 | 7 | 5 | 3 | 1 | 3 | 5 | 7 | 9 | C11 |
| C9 | 9 | 7 | 5 | 3 | 1 | 3 | 5 | 7 | 9 | C12 |
| C9 | 9 | 7 | 5 | 3 | 1 | 3 | 5 | 7 | 9 | C13 |
| C10 | 9 | 7 | 5 | 3 | 1 | 3 | 5 | 7 | 9 | C14 |
| C10 | 9 | 7 | 5 | 3 | 1 | 3 | 5 | 7 | 9 | C15 |
| C10 | 9 | 7 | 5 | 3 | 1 | 3 | 5 | 7 | 9 | C16 |
| C10 | 9 | 7 | 5 | 3 | 1 | 3 | 5 | 7 | 9 | C17 |
| C10 | 9 | 7 | 5 | 3 | 1 | 3 | 5 | 7 | 9 | C18 |
| C10 | 9 | 7 | 5 | 3 | 1 | 3 | 5 | 7 | 9 | C19 |
| C10 | 9 | 7 | 5 | 3 | 1 | 3 | 5 | 7 | 9 | C20 |
| C10 | 9 | 7 | 5 | 3 | 1 | 3 | 5 | 7 | 9 | C21 |
| C10 | 9 | 7 | 5 | 3 | 1 | 3 | 5 | 7 | 9 | C22 |
| C10 | 9 | 7 | 5 | 3 | 1 | 3 | 5 | 7 | 9 | C10 |
| C10 | 9 | 7 | 5 | 3 | 1 | 3 | 5 | 7 | 9 | C11 |
| C10 | 9 | 7 | 5 | 3 | 1 | 3 | 5 | 7 | 9 | C12 |
| C11 | 9 | 7 | 5 | 3 | 1 | 3 | 5 | 7 | 9 | C13 |
| C11 | 9 | 7 | 5 | 3 | 1 | 3 | 5 | 7 | 9 | C14 |
| C11 | 9 | 7 | 5 | 3 | 1 | 3 | 5 | 7 | 9 | C15 |
| C11 | 9 | 7 | 5 | 3 | 1 | 3 | 5 | 7 | 9 | C16 |
| C11 | 9 | 7 | 5 | 3 | 1 | 3 | 5 | 7 | 9 | C17 |
| C11 | 9 | 7 | 5 | 3 | 1 | 3 | 5 | 7 | 9 | C18 |
| C11 | 9 | 7 | 5 | 3 | 1 | 3 | 5 | 7 | 9 | C19 |
| C11 | 9 | 7 | 5 | 3 | 1 | 3 | 5 | 7 | 9 | C20 |
| C11 | 9 | 7 | 5 | 3 | 1 | 3 | 5 | 7 | 9 | C21 |
| C11 | 9 | 7 | 5 | 3 | 1 | 3 | 5 | 7 | 9 | C22 |
| C11 | 9 | 7 | 5 | 3 | 1 | 3 | 5 | 7 | 9 | C11 |
| C12 | 9 | 7 | 5 | 3 | 1 | 3 | 5 | 7 | 9 | C12 |
| C12 | 9 | 7 | 5 | 3 | 1 | 3 | 5 | 7 | 9 | C13 |
| C12 | 9 | 7 | 5 | 3 | 1 | 3 | 5 | 7 | 9 | C14 |
| C12 | 9 | 7 | 5 | 3 | 1 | 3 | 5 | 7 | 9 | C15 |
| C12 | 9 | 7 | 5 | 3 | 1 | 3 | 5 | 7 | 9 | C16 |
| C12 | 9 | 7 | 5 | 3 | 1 | 3 | 5 | 7 | 9 | C17 |
| C12 | 9 | 7 | 5 | 3 | 1 | 3 | 5 | 7 | 9 | C18 |
| C12 | 9 | 7 | 5 | 3 | 1 | 3 | 5 | 7 | 9 | C19 |
| C12 | 9 | 7 | 5 | 3 | 1 | 3 | 5 | 7 | 9 | C20 |
| C12 | 9 | 7 | 5 | 3 | 1 | 3 | 5 | 7 | 9 | C21 |
| C13 | 9 | 7 | 5 | 3 | 1 | 3 | 5 | 7 | 9 | C22 |
| C13 | 9 | 7 | 5 | 3 | 1 | 3 | 5 | 7 | 9 | C12 |
| C13 | 9 | 7 | 5 | 3 | 1 | 3 | 5 | 7 | 9 | C13 |
| C13 | 9 | 7 | 5 | 3 | 1 | 3 | 5 | 7 | 9 | C14 |
| C13 | 9 | 7 | 5 | 3 | 1 | 3 | 5 | 7 | 9 | C15 |
| C13 | 9 | 7 | 5 | 3 | 1 | 3 | 5 | 7 | 9 | C16 |
| C13 | 9 | 7 | 5 | 3 | 1 | 3 | 5 | 7 | 9 | C17 |
| C13 | 9 | 7 | 5 | 3 | 1 | 3 | 5 | 7 | 9 | C18 |
| C13 | 9 | 7 | 5 | 3 | 1 | 3 | 5 | 7 | 9 | C19 |
| C14 | 9 | 7 | 5 | 3 | 1 | 3 | 5 | 7 | 9 | C20 |

**Table 5.** (continued)

| C14 | 9 | 7 | 5 | 3 | 1 | 3 | 5 | 7 | 9 | C21 |
| --- | --- | --- | --- | --- | --- | --- | --- | --- | --- | --- |
| C14 | 9 | 7 | 5 | 3 | 1 | 3 | 5 | 7 | 9 | C22 |
| C14 | 9 | 7 | 5 | 3 | 1 | 3 | 5 | 7 | 9 | C13 |
| C14 | 9 | 7 | 5 | 3 | 1 | 3 | 5 | 7 | 9 | C14 |
| C14 | 9 | 7 | 5 | 3 | 1 | 3 | 5 | 7 | 9 | C15 |
| C14 | 9 | 7 | 5 | 3 | 1 | 3 | 5 | 7 | 9 | C16 |
| C14 | 9 | 7 | 5 | 3 | 1 | 3 | 5 | 7 | 9 | C17 |
| C15 | 9 | 7 | 5 | 3 | 1 | 3 | 5 | 7 | 9 | C18 |
| C15 | 9 | 7 | 5 | 3 | 1 | 3 | 5 | 7 | 9 | C19 |
| C15 | 9 | 7 | 5 | 3 | 1 | 3 | 5 | 7 | 9 | C20 |
| C15 | 9 | 7 | 5 | 3 | 1 | 3 | 5 | 7 | 9 | C21 |
| C15 | 9 | 7 | 5 | 3 | 1 | 3 | 5 | 7 | 9 | C22 |
| C15 | 9 | 7 | 5 | 3 | 1 | 3 | 5 | 7 | 9 | C14 |
| C15 | 9 | 7 | 5 | 3 | 1 | 3 | 5 | 7 | 9 | C15 |
| C16 | 9 | 7 | 5 | 3 | 1 | 3 | 5 | 7 | 9 | C16 |
| C16 | 9 | 7 | 5 | 3 | 1 | 3 | 5 | 7 | 9 | C17 |
| C16 | 9 | 7 | 5 | 3 | 1 | 3 | 5 | 7 | 9 | C18 |
| C16 | 9 | 7 | 5 | 3 | 1 | 3 | 5 | 7 | 9 | C19 |
| C16 | 9 | 7 | 5 | 3 | 1 | 3 | 5 | 7 | 9 | C20 |
| C16 | 9 | 7 | 5 | 3 | 1 | 3 | 5 | 7 | 9 | C21 |
| C17 | 9 | 7 | 5 | 3 | 1 | 3 | 5 | 7 | 9 | C22 |
| C17 | 9 | 7 | 5 | 3 | 1 | 3 | 5 | 7 | 9 | C15 |
| C17 | 9 | 7 | 5 | 3 | 1 | 3 | 5 | 7 | 9 | C16 |
| C17 | 9 | 7 | 5 | 3 | 1 | 3 | 5 | 7 | 9 | C17 |
| C17 | 9 | 7 | 5 | 3 | 1 | 3 | 5 | 7 | 9 | C18 |
| C18 | 9 | 7 | 5 | 3 | 1 | 3 | 5 | 7 | 9 | C19 |
| C18 | 9 | 7 | 5 | 3 | 1 | 3 | 5 | 7 | 9 | C20 |
| C18 | 9 | 7 | 5 | 3 | 1 | 3 | 5 | 7 | 9 | C21 |
| C18 | 9 | 7 | 5 | 3 | 1 | 3 | 5 | 7 | 9 | C22 |
| C19 | 9 | 7 | 5 | 3 | 1 | 3 | 5 | 7 | 9 | C16 |
| C19 | 9 | 7 | 5 | 3 | 1 | 3 | 5 | 7 | 9 | C17 |
| C19 | 9 | 7 | 5 | 3 | 1 | 3 | 5 | 7 | 9 | C18 |
| C20 | 9 | 7 | 5 | 3 | 1 | 3 | 5 | 7 | 9 | C19 |
| C20 | 9 | 7 | 5 | 3 | 1 | 3 | 5 | 7 | 9 | C20 |
| C21 | 9 | 7 | 5 | 3 | 1 | 3 | 5 | 7 | 9 | C21 |

(C1): Removal performance; (C2): Reliability and durability; (C3): Simplicity; (C4): Ease of planned maintenance; (C5): Ease of operation and routine maintenance; (C6): Ease of construction; (C7): Usage of various chemical; (C8): Major operational consumables; (C9): Proven/establishment technology; (C10): Generated wastes; (C11): Treatment/management requirements of wastes; (C12): Water efficiency; (C13): Security of supply; (C14): Asset life; (C15): Availability of technology; (C16): Pretreatment requirements; (C17): By product/metabolite formation; (C18): Suitability of application: (C19): Additional treatments; (C20): Environmental impacts; (C21): Use of natural resources; (C22): Safety risk (C22)

(A1): Conventional treatments; (A2): Adsorption oxidation; (A3): Advanced oxidation; (A4): Membrane Filtration; (A5): Hybrid Processes.

**Appendix 2 Pairwise comparison matrix of alternatives in terms of criteria**

**Table 6.** Pairwise comparison of alternatives and consistency ratio for C1 criterion

| **C1** | | | | | | | |
| --- | --- | --- | --- | --- | --- | --- | --- |
|  | A1 | A2 | A3 | | A4 | A5 | A5 |
| A1 | 1.00 | 0.17 | 0.50 | | 0.13 | 0.14 | 0.33 |
| A2 | 6.00 | 1.00 | 5.00 | | 0.33 | 0.50 | 4.00 |
| A3 | 2.00 | 0.20 | 1.00 | | 0.14 | 0.17 | 0.50 |
| A4 | 8.00 | 3.00 | 7.00 | | 1.00 | 2.00 | 6.00 |
| A5 | 7.00 | 2.00 | 6.00 | | 0.50 | 1.00 | 5.00 |
| A6 | 3.00 | 0.25 | 2.00 | | 0.17 | 0.20 | 1.00 |
| CI: | | | | 0.04 | | | |
| RI: | | | | 1.24 | | | |
| CR (should be <0.01): | | | | 0.032 | | | |

**Table 7.** Pairwise comparison of alternatives and consistency ratio for C2 criterion

| C2 | | | | | | | |
| --- | --- | --- | --- | --- | --- | --- | --- |
|  | A1 | A2 | A3 | | A4 | A5 | A6 |
| A1 | 1.00 | 2.00 | 2.00 | | 2.00 | 0.33 | 0.50 |
| A2 | 0.50 | 1.00 | 1.00 | | 1.00 | 0.25 | 0.33 |
| A3 | 0.50 | 1.00 | 1.00 | | 1.00 | 0.25 | 0.33 |
| A4 | 0.50 | 1.00 | 1.00 | | 1.00 | 0.25 | 0.33 |
| A5 | 3.00 | 4.00 | 4.00 | | 4.00 | 1.00 | 2.00 |
| A6 | 2.00 | 3.00 | 3.00 | | 3.00 | 0.50 | 1.00 |
| CI: | | | | 0.01 | | | |
| RI: | | | | 1.24 | | | |
| CR (should be <0.01): | | | | 0.006 | | | |

**Table 8.** Pairwise comparison of alternatives and consistency ratio for C3 criterion

| **C3** | | | | | | | |
| --- | --- | --- | --- | --- | --- | --- | --- |
|  | A1 | A2 | A3 | | A4 | A5 | A6 |
| A1 | 1.00 | 5.00 | 2.00 | | 3.00 | 5.00 | 4.00 |
| A2 | 0.20 | 1.00 | 0.25 | | 0.33 | 1.00 | 0.50 |
| A3 | 0.50 | 4.00 | 1.00 | | 2.00 | 4.00 | 3.00 |
| A4 | 0.33 | 3.00 | 0.50 | | 1.00 | 3.00 | 2.00 |
| A5 | 0.20 | 1.00 | 0.25 | | 0.33 | 1.00 | 0.50 |
| A6 | 0.25 | 2.00 | 0.33 | | 0.50 | 2.00 | 1.00 |
| CI: | | | | 0.02 | | | |
| RI: | | | | 1.24 | | | |
| CR (should be <0.01): | | | | 0.013 | | | |

**Table 9.** Pairwise comparison of alternatives and consistency ratio for C4 criterion

| **C4** | | | | | | | |
| --- | --- | --- | --- | --- | --- | --- | --- |
|  | A1 | A2 | A3 | | A4 | A5 | A6 |
| A1 | 1.00 | 5.00 | 2.00 | | 3.00 | 6.00 | 4.00 |
| A2 | 0.20 | 1.00 | 0.25 | | 0.33 | 2.00 | 0.50 |
| A3 | 0.50 | 4.00 | 1.00 | | 2.00 | 5.00 | 3.00 |
| A4 | 0.33 | 3.00 | 0.50 | | 1.00 | 4.00 | 2.00 |
| A5 | 0.17 | 0.50 | 0.20 | | 0.25 | 1.00 | 0.33 |
| A6 | 0.25 | 2.00 | 0.33 | | 0.50 | 3.00 | 1.00 |
| CI: | | | | 0.02 | | | |
| RI: | | | | 1.24 | | | |
| CR (should be <0.01): | | | | 0.020 | | | |

**Table 10.** Pairwise comparison of alternatives and consistency ratio for C5 criterion

| **C5** | | | | | | | |
| --- | --- | --- | --- | --- | --- | --- | --- |
|  | A1 | A2 | A3 | | A4 | A5 | A6 |
| A1 | 1.00 | 2.00 | 0.33 | | 0.50 | 4.00 | 3.00 |
| A2 | 0.50 | 1.00 | 0.25 | | 0.33 | 3.00 | 2.00 |
| A3 | 3.00 | 4.00 | 1.00 | | 2.00 | 6.00 | 5.00 |
| A4 | 2.00 | 3.00 | 0.50 | | 1.00 | 5.00 | 4.00 |
| A5 | 0.25 | 0.33 | 0.17 | | 0.20 | 1.00 | 0.50 |
| A6 | 0.33 | 0.50 | 0.20 | | 0.25 | 2.00 | 1.00 |
| CI: | | | | 0.02 | | | |
| RI: | | | | 1.24 | | | |
| CR (should be <0.01): | | | | 0.020 | | | |

**Table 11.** Pairwise comparison of alternatives and consistency ratio for C6 criterion

| **C6** | | | | | | | |
| --- | --- | --- | --- | --- | --- | --- | --- |
|  | A1 | A2 | A3 | | A4 | A5 | A5 |
| A1 | 1.00 | 4.00 | 3.00 | | 3.00 | 4.00 | 2.00 |
| A2 | 0.25 | 1.00 | 0.50 | | 0.50 | 1.00 | 0.33 |
| A3 | 0.33 | 2.00 | 1.00 | | 1.00 | 2.00 | 0.50 |
| A4 | 0.33 | 2.00 | 1.00 | | 1.00 | 2.00 | 0.50 |
| A5 | 0.25 | 1.00 | 0.50 | | 0.50 | 1.00 | 0.33 |
| A6 | 0.50 | 3.00 | 2.00 | | 2.00 | 3.00 | 1.00 |
| CI: | | | | 0.01 | | | |
| RI: | | | | 1.24 | | | |
| CR (should be <0.01): | | | | 0.007 | | | |

**Table 12.** Pairwise comparison of alternatives and consistency ratio for C7 criterion

| **C7** | | | | | | | |
| --- | --- | --- | --- | --- | --- | --- | --- |
|  | A1 | A2 | A3 | | A4 | A5 | A6 |
| A1 | 1.00 | 3.00 | 3.00 | | 0.50 | 3.00 | 2.00 |
| A2 | 0.33 | 1.00 | 1.00 | | 0.25 | 1.00 | 0.50 |
| A3 | 0.33 | 1.00 | 1.00 | | 0.25 | 1.00 | 0.50 |
| A4 | 2.00 | 4.00 | 4.00 | | 1.00 | 4.00 | 3.00 |
| A5 | 0.33 | 1.00 | 1.00 | | 0.25 | 1.00 | 0.50 |
| A6 | 0.50 | 2.00 | 2.00 | | 0.33 | 2.00 | 1.00 |
| CI: | | | | 0.01 | | | |
| RI: | | | | 1.24 | | | |
| CR (should be <0.01): | | | | 0.006 | | | |

**Table 13.** Pairwise comparison of alternatives and consistency ratio for C8 criterion

| **C8** | | | | | | | |
| --- | --- | --- | --- | --- | --- | --- | --- |
|  | A1 | A2 | A3 | | A4 | A5 | A6 |
| A1 | 1.00 | 4.00 | 3.00 | | 4.00 | 1.00 | 2.00 |
| A2 | 0.25 | 1.00 | 0.50 | | 0.50 | 0.25 | 0.33 |
| A3 | 0.33 | 2.00 | 1.00 | | 1.00 | 0.33 | 0.50 |
| A4 | 0.25 | 2.00 | 1.00 | | 1.00 | 0.33 | 0.50 |
| A5 | 1.00 | 4.00 | 3.00 | | 3.00 | 1.00 | 2.00 |
| A6 | 0.50 | 3.00 | 2.00 | | 2.00 | 0.50 | 1.00 |
| CI: | | | | 0.01 | | | |
| RI: | | | | 1.24 | | | |
| CR (should be <0.01): | | | | 0.008 | | | |

**Table 14.** Pairwise comparison of alternatives and consistency ratio for C9 criterion

| **C9** | | | | | | | |
| --- | --- | --- | --- | --- | --- | --- | --- |
|  | A1 | A2 | A3 | | A4 | A5 | A6 |
| A1 | 1.00 | 0.50 | 2.00 | | 2.00 | 0.50 | 1.00 |
| A2 | 2.00 | 1.00 | 3.00 | | 3.00 | 1.00 | 2.00 |
| A3 | 0.50 | 0.33 | 1.00 | | 1.00 | 0.33 | 0.50 |
| A4 | 0.50 | 0.33 | 1.00 | | 1.00 | 0.33 | 0.50 |
| A5 | 2.00 | 1.00 | 3.00 | | 3.00 | 1.00 | 2.00 |
| A6 | 1.00 | 0.50 | 2.00 | | 2.00 | 0.50 | 1.00 |
| CI: | | | | 0.004 | | | |
| RI: | | | | 1.24 | | | |
| CR (should be <0.01): | | | | 0.003 | | | |

**Table 15.** Pairwise comparison of alternatives and consistency ratio for C10 criterion

| **C10** | | | | | | | |
| --- | --- | --- | --- | --- | --- | --- | --- |
|  | A1 | A2 | A3 | | A4 | A5 | A6 |
| A1 | 1.00 | 0.33 | 0.33 | | 0.33 | 1.00 | 0.50 |
| A2 | 3.00 | 1.00 | 1.00 | | 1.00 | 3.00 | 2.00 |
| A3 | 3.00 | 1.00 | 1.00 | | 1.00 | 3.00 | 2.00 |
| A4 | 3.00 | 1.00 | 1.00 | | 1.00 | 3.00 | 2.00 |
| A5 | 1.00 | 0.33 | 0.33 | | 0.33 | 1.00 | 0.50 |
| A6 | 2.00 | 0.50 | 0.50 | | 0.50 | 2.00 | 1.00 |
| CI: | | | | 0.03 | | | |
| RI: | | | | 1.24 | | | |
| CR (should be <0.01): | | | | 0.002 | | | |

**Table 16.** Pairwise comparison of alternatives and consistency ratio for C11 criterion

| **C11** | | | | | | | |
| --- | --- | --- | --- | --- | --- | --- | --- |
|  | A1 | A2 | A3 | | A4 | A5 | A6 |
| A1 | 1.00 | 1.00 | 3.00 | | 3.00 | 1.00 | 2.00 |
| A2 | 1.00 | 1.00 | 3.00 | | 3.00 | 1.00 | 2.00 |
| A3 | 0.33 | 0.33 | 1.00 | | 0.33 | 0.33 | 0.50 |
| A4 | 0.33 | 0.33 | 3.00 | | 1.00 | 0.33 | 0.50 |
| A5 | 1.00 | 1.00 | 3.00 | | 3.00 | 1.00 | 2.00 |
| A6 | 0.50 | 0.50 | 2.00 | | 2.00 | 0.50 | 1.00 |
| CI: | | | | 0.03 | | | |
| RI: | | | | 1.24 | | | |
| CR (should be <0.01): | | | | 0.026 | | | |

**Table 17.** Pairwise comparison of alternatives and consistency ratio for C12 criterion

| **C12** | | | | | | | |
| --- | --- | --- | --- | --- | --- | --- | --- |
|  | A1 | A2 | A3 | | A4 | A5 | A6 |
| A1 | 1.00 | 0.33 | 0.33 | | 0.33 | 1.00 | 0.50 |
| A2 | 3.00 | 1.00 | 1.00 | | 1.00 | 3.00 | 2.00 |
| A3 | 3.00 | 1.00 | 1.00 | | 1.00 | 3.00 | 2.00 |
| A4 | 3.00 | 1.00 | 1.00 | | 1.00 | 3.00 | 2.00 |
| A5 | 1.00 | 0.33 | 0.33 | | 0.33 | 1.00 | 0.50 |
| A6 | 2.00 | 0.50 | 0.50 | | 0.50 | 2.00 | 1.00 |
| CI: | | | | 0.003 | | | |
| RI: | | | | 1.24 | | | |
| CR (should be <0.01): | | | | 0.002 | | | |

**Table 18.** Pairwise comparison of alternatives and consistency ratio for C13 criterion

| **C13** | | | | | | | |
| --- | --- | --- | --- | --- | --- | --- | --- |
|  | A1 | A2 | A3 | | A4 | A5 | A6 |
| A1 | 1.00 | 1.00 | 3.00 | | 3.00 | 1.00 | 2.00 |
| A2 | 1.00 | 1.00 | 3.00 | | 3.00 | 1.00 | 2.00 |
| A3 | 0.33 | 0.33 | 1.00 | | 1.00 | 0.33 | 0.50 |
| A4 | 0.33 | 0.33 | 1.00 | | 1.00 | 0.33 | 0.50 |
| A5 | 1.00 | 1.00 | 3.00 | | 3.00 | 1.00 | 2.00 |
| A6 | 0.50 | 0.50 | 2.00 | | 2.00 | 0.50 | 1.00 |
| CI: | | | | 0.003 | | | |
| RI: | | | | 1.24 | | | |
| CR (should be <0.01): | | | | 0.002 | | | |

**Table 19.** Pairwise comparison of alternatives and consistency ratio for C14 criterion

| **C14** | | | | | | | |
| --- | --- | --- | --- | --- | --- | --- | --- |
|  | A1 | A2 | A3 | | A4 | A5 | A6 |
| A1 | 1.00 | 2.00 | 2.00 | | 2.00 | 2.00 | 2.00 |
| A2 | 0.50 | 1.00 | 1.00 | | 1.00 | 1.00 | 1.00 |
| A3 | 0.50 | 1.00 | 1.00 | | 1.00 | 1.00 | 1.00 |
| A4 | 0.50 | 1.00 | 1.00 | | 1.00 | 1.00 | 1.00 |
| A5 | 0.50 | 1.00 | 1.00 | | 1.00 | 1.00 | 1.00 |
| A6 | 0.50 | 1.00 | 1.00 | | 1.00 | 1.00 | 1.00 |
| CI: | | | | 0.00 | | | |
| RI: | | | | 1.24 | | | |
| CR (should be <0.01): | | | | 0.000 | | | |

**Table 20.** Pairwise comparison of alternatives and consistency ratio for C15 criterion

| **C15** | | | | | | | |
| --- | --- | --- | --- | --- | --- | --- | --- |
|  | A1 | A2 | A3 | | A4 | A5 | A6 |
| A1 | 1.00 | 0.33 | 0.50 | | 0.13 | 0.17 | 0.20 |
| A2 | 3.00 | 1.00 | 2.00 | | 0.17 | 0.25 | 0.33 |
| A3 | 2.00 | 0.50 | 1.00 | | 0.14 | 0.20 | 0.25 |
| A4 | 8.00 | 6.00 | 7.00 | | 1.00 | 3.00 | 4.00 |
| A5 | 6.00 | 4.00 | 5.00 | | 0.33 | 1.00 | 2.00 |
| A6 | 5.00 | 3.00 | 4.00 | | 0.25 | 0.50 | 1.00 |
| CI: | | | | 0.04 | | | |
| RI: | | | | 1.24 | | | |
| CR (should be <0.01): | | | | 0.035 | | | |

**Table 21.** Pairwise comparison of alternatives and consistency ratio for C16 criterion

| **C16** | | | | | | | |
| --- | --- | --- | --- | --- | --- | --- | --- |
|  | A1 | A2 | A3 | | A4 | A5 | A6 |
| A1 | 1.00 | 2.00 | 4.00 | | 4.00 | 3.00 | 2.00 |
| A2 | 0.50 | 1.00 | 3.00 | | 3.00 | 2.00 | 1.00 |
| A3 | 0.25 | 0.33 | 1.00 | | 1.00 | 0.50 | 0.33 |
| A4 | 0.25 | 0.33 | 1.00 | | 1.00 | 0.50 | 0.33 |
| A5 | 0.33 | 0.50 | 2.00 | | 2.00 | 1.00 | 0.50 |
| A6 | 0.50 | 1.00 | 3.00 | | 3.00 | 2.00 | 1.00 |
| CI: | | | | 0.01 | | | |
| RI: | | | | 1.24 | | | |
| CR (should be <0.01): | | | | 0.007 | | | |

**Table 22.** Pairwise comparison of alternatives and consistency ratio for C17 criterion

| **C17** | | | | | | | |
| --- | --- | --- | --- | --- | --- | --- | --- |
|  | A1 | A2 | A3 | | A4 | A5 | A6 |
| A1 | 1.00 | 1.00 | 0.20 | | 1.00 | 1.00 | 0.33 |
| A2 | 1.00 | 1.00 | 0.20 | | 1.00 | 1.00 | 0.33 |
| A3 | 5.00 | 5.00 | 1.00 | | 5.00 | 5.00 | 3.00 |
| A4 | 1.00 | 1.00 | 0.20 | | 1.00 | 1.00 | 0.33 |
| A5 | 1.00 | 1.00 | 0.20 | | 1.00 | 1.00 | 0.33 |
| A6 | 3.00 | 3.00 | 0.33 | | 3.00 | 3.00 | 1.00 |
| CI: | | | | 0.01 | | | |
| RI: | | | | 1.24 | | | |
| CR (should be <0.01): | | | | 0.006 | | | |

**Table 23.** Pairwise comparison of alternatives and consistency ratio for C18 criterion

| **C18** | | | | | | | |
| --- | --- | --- | --- | --- | --- | --- | --- |
|  | A1 | A2 | A3 | | A4 | A5 | A6 |
| A1 | 1.00 | 0.14 | 0.50 | | 0.13 | 0.17 | 0.33 |
| A2 | 7.00 | 1.00 | 6.00 | | 0.50 | 2.00 | 5.00 |
| A3 | 2.00 | 0.17 | 1.00 | | 0.14 | 0.20 | 0.50 |
| A4 | 8.00 | 2.00 | 7.00 | | 1.00 | 3.00 | 6.00 |
| A5 | 6.00 | 0.50 | 5.00 | | 0.33 | 1.00 | 4.00 |
| A6 | 3.00 | 0.20 | 2.00 | | 0.17 | 0.25 | 1.00 |
| CI: | | | | 0.04 | | | |
| RI: | | | | 1.24 | | | |
| CR (should be <0.01): | | | | 0.032 | | | |

**Table 24.** Pairwise comparison of alternatives and consistency ratio for C19 criterion

| **C19** | | | | | | | |
| --- | --- | --- | --- | --- | --- | --- | --- |
|  | A1 | A2 | A3 | | A4 | A5 | A6 |
| A1 | 1.00 | 0.13 | 1.00 | | 0.14 | 0.17 | 0.17 |
| A2 | 8.00 | 1.00 | 7.00 | | 0.50 | 2.00 | 2.00 |
| A3 | 1.00 | 0.14 | 1.00 | | 0.13 | 0.17 | 0.17 |
| A4 | 7.00 | 2.00 | 8.00 | | 1.00 | 3.00 | 3.00 |
| A5 | 6.00 | 0.50 | 6.00 | | 0.33 | 1.00 | 1.00 |
| A6 | 6.00 | 0.50 | 6.00 | | 0.33 | 1.00 | 1.00 |
| CI: | | | | 0.03 | | | |
| RI: | | | | 1.24 | | | |
| CR (should be <0.01): | | | | 0.024 | | | |

**Table 25.** Pairwise comparison of alternatives and consistency ratio for C20 criterion

| **C20** | | | | | | | |
| --- | --- | --- | --- | --- | --- | --- | --- |
|  | A1 | A2 | A3 | | A4 | A5 | A6 |
| A1 | 1.00 | 2.00 | 4.00 | | 4.00 | 4.00 | 3.00 |
| A2 | 0.50 | 1.00 | 3.00 | | 3.00 | 3.00 | 2.00 |
| A3 | 0.25 | 0.33 | 1.00 | | 1.00 | 1.00 | 0.50 |
| A4 | 0.25 | 0.33 | 1.00 | | 1.00 | 1.00 | 0.50 |
| A5 | 0.25 | 0.33 | 1.00 | | 1.00 | 1.00 | 0.50 |
| A6 | 0.33 | 0.50 | 2.00 | | 2.00 | 2.00 | 1.00 |
| CI: | | | | 0.01 | | | |
| RI: | | | | 1.24 | | | |
| CR (should be <0.01): | | | | 0.006 | | | |

**Table 26.** Pairwise comparison of alternatives and consistency ratio for C21 criterion

| **C21** | | | | | | | |
| --- | --- | --- | --- | --- | --- | --- | --- |
|  | A1 | A2 | A3 | | A4 | A5 | A6 |
| A1 | 1.00 | 0.13 | 1.00 | | 1.00 | 1.00 | 0.14 |
| A2 | 8.00 | 1.00 | 8.00 | | 8.00 | 8.00 | 2.00 |
| A3 | 1.00 | 0.13 | 1.00 | | 1.00 | 1.00 | 0.14 |
| A4 | 1.00 | 0.13 | 1.00 | | 1.00 | 1.00 | 0.14 |
| A5 | 1.00 | 0.13 | 1.00 | | 1.00 | 1.00 | 0.14 |
| A6 | 7.00 | 0.50 | 7.00 | | 7.00 | 7.00 | 1.00 |
| CI: | | | | 0.01 | | | |
| RI: | | | | 1.24 | | | |
| CR (should be <0.01): | | | | 0.006 | | | |

**Table 27.** Pairwise comparison of alternatives and consistency ratio for C22 criterion

| **C22** | | | | | | | |
| --- | --- | --- | --- | --- | --- | --- | --- |
|  | A1 | A2 | A3 | | A4 | A5 | A6 |
| A1 | 1.00 | 2.00 | 0.14 | | 0.17 | 0.50 | 0.20 |
| A2 | 0.50 | 1.00 | 0.13 | | 0.14 | 0.33 | 0.17 |
| A3 | 7.00 | 8.00 | 1.00 | | 2.00 | 5.00 | 3.00 |
| A4 | 6.00 | 7.00 | 0.50 | | 1.00 | 5.00 | 2.00 |
| A5 | 2.00 | 3.00 | 0.20 | | 0.20 | 1.00 | 0.25 |
| A6 | 5.00 | 6.00 | 0.33 | | 0.50 | 4.00 | 1.00 |
| CI: | | | | 0.04 | | | |
| RI: | | | | 1.24 | | | |
| CR (should be <0.01): | | | | 0.032 | | | |

**Appendix 3 Ranking of alternatives specific to individual criteria**

**Table 28.** Ranking of alternatives specific to individual criteria

|  | **C1** | **C2** | **C3** | **C4** | **C5** | **C6** | **C7** | **C8** | **C9** | **C10** | **C11** | **C12** | **C13** | **C14** | **C15** | **C16** | **C17** | **C18** | **C19** | **C20** | **C21** | **C22** |
| --- | --- | --- | --- | --- | --- | --- | --- | --- | --- | --- | --- | --- | --- | --- | --- | --- | --- | --- | --- | --- | --- | --- |
| **A1** | 6 | 3 | 1 | 1 | 3 | 1 | 2 | 1 | 3 | 5 | 1 | 5 | 1 | 1 | 6 | 1 | 3 | 6 | 5 | 1 | 3 | 5 |
| **A2** | 3 | 4 | 5 | 5 | 4 | 5 | 4 | 6 | 1 | 1 | 1 | 1 | 1 | 2 | 4 | 2 | 3 | 2 | 2 | 2 | 1 | 6 |
| **A3** | 5 | 4 | 2 | 2 | 1 | 3 | 4 | 4 | 5 | 1 | 6 | 1 | 5 | 2 | 5 | 5 | 1 | 5 | 6 | 4 | 3 | 1 |
| **A4** | 1 | 4 | 3 | 3 | 2 | 3 | 1 | 5 | 5 | 1 | 5 | 1 | 5 | 2 | 1 | 5 | 3 | 1 | 1 | 4 | 3 | 2 |
| **A5** | 2 | 1 | 5 | 6 | 6 | 5 | 4 | 2 | 1 | 5 | 1 | 5 | 1 | 2 | 2 | 4 | 3 | 3 | 3 | 4 | 3 | 4 |
| **A6** | 4 | 2 | 4 | 4 | 5 | 2 | 3 | 3 | 3 | 4 | 4 | 4 | 4 | 2 | 3 | 2 | 2 | 4 | 3 | 3 | 2 | 3 |

(C1): Removal performance; (C2): Reliability and durability; (C3): Simplicity; (C4): Ease of planned maintenance; (C5): Ease of operation and routine maintenance; (C6): Ease of construction; (C7): Usage of various chemical; (C8): Major operational consumables; (C9): Proven/establishment technology; (C10): Generated wastes; (C11): Treatment/management requirements of wastes; (C12): Water efficiency; (C13): Security of supply; (C14): Asset life; (C15): Availability of technology; (C16): Pretreatment requirements; (C17): By product/metabolite formation; (C18): Suitability of application: (C19): Additional treatments; (C20): Environmental impacts; (C21): Use of natural resources; (C22): Safety risk (C22)

(A1): Conventional treatments; (A2): Adsorption oxidation; (A3): Advanced oxidation; (A4): Membrane Filtration; (A5): Hybrid Processes.
